# A transposable element-derived regulatory variation in *RsOFP2.3* underlies morphological diversification of radish taproots through the conserved OVATE family protein-TONNEAU1-recruiting motif module

**DOI:** 10.1093/hr/uhag127

**Published:** 2026-04-07

**Authors:** Yanping Wang, Xiangyu Wu, Yuanting Pang, Dingzhu Hua, Ting Chi, Ailing Ge, Tongbing Su, Qingbiao Wang, Li Zhang

**Affiliations:** State Key Laboratory of Vegetable Biobreeding, Beijing Vegetable Research Center, National Engineering Research Center for Vegetables, Beijing Academy of Agriculture and Forestry Sciences, Beijing 100097, China; Key Laboratory of Biology and Genetics Improvement of Horticultural Crops (North China), Beijing 100097, China; State Key Laboratory of Vegetable Biobreeding, Beijing Vegetable Research Center, National Engineering Research Center for Vegetables, Beijing Academy of Agriculture and Forestry Sciences, Beijing 100097, China; Key Laboratory of Biology and Genetics Improvement of Horticultural Crops (North China), Beijing 100097, China; State Key Laboratory of Vegetable Biobreeding, Beijing Vegetable Research Center, National Engineering Research Center for Vegetables, Beijing Academy of Agriculture and Forestry Sciences, Beijing 100097, China; Key Laboratory of Biology and Genetics Improvement of Horticultural Crops (North China), Beijing 100097, China; College of Horticulture, China Agricultural University, Beijing 100193, China; State Key Laboratory of Vegetable Biobreeding, Beijing Vegetable Research Center, National Engineering Research Center for Vegetables, Beijing Academy of Agriculture and Forestry Sciences, Beijing 100097, China; Key Laboratory of Biology and Genetics Improvement of Horticultural Crops (North China), Beijing 100097, China; College of Horticulture, China Agricultural University, Beijing 100193, China; State Key Laboratory of Vegetable Biobreeding, Beijing Vegetable Research Center, National Engineering Research Center for Vegetables, Beijing Academy of Agriculture and Forestry Sciences, Beijing 100097, China; Key Laboratory of Biology and Genetics Improvement of Horticultural Crops (North China), Beijing 100097, China; College of Agriculture, Guangxi University, Nanning 530004, China; State Key Laboratory of Vegetable Biobreeding, Beijing Vegetable Research Center, National Engineering Research Center for Vegetables, Beijing Academy of Agriculture and Forestry Sciences, Beijing 100097, China; Key Laboratory of Biology and Genetics Improvement of Horticultural Crops (North China), Beijing 100097, China; College of Horticultural Science and Technology, Hebei Normal University of Science and Technology, Qinhuangdao 066600, China; State Key Laboratory of Vegetable Biobreeding, Beijing Vegetable Research Center, National Engineering Research Center for Vegetables, Beijing Academy of Agriculture and Forestry Sciences, Beijing 100097, China; Key Laboratory of Biology and Genetics Improvement of Horticultural Crops (North China), Beijing 100097, China; State Key Laboratory of Vegetable Biobreeding, Beijing Vegetable Research Center, National Engineering Research Center for Vegetables, Beijing Academy of Agriculture and Forestry Sciences, Beijing 100097, China; Key Laboratory of Biology and Genetics Improvement of Horticultural Crops (North China), Beijing 100097, China; State Key Laboratory of Vegetable Biobreeding, Beijing Vegetable Research Center, National Engineering Research Center for Vegetables, Beijing Academy of Agriculture and Forestry Sciences, Beijing 100097, China; Key Laboratory of Biology and Genetics Improvement of Horticultural Crops (North China), Beijing 100097, China

## Abstract

Radish (*Raphanus sativus* L.) represents a major root vegetable crop exhibiting remarkable variation in taproot morphology, yet the underlying genetic and molecular mechanisms remain poorly understood. Through a structural variant (SV)-based genome-wide association study (GWAS), we identified *RsOFP2.3*, encoding an OVATE Family Protein (OFP), as a major determinant of fleshy taproot shape. Comprehensive expression profiling and RNA *in situ* hybridization revealed that *RsOFP2.3* is broadly expressed, with the highest transcript accumulation in cambial tissues. Notably, *RsOFP2.3* expression was markedly higher in the round-rooted accession than in the long-rooted one. Population-wide analysis showed that *RsOFP2.3* expression was negatively correlated with taproot length and shape index, but positively correlated with taproot width. A 312-bp transposable element (TE) insertion in the *RsOFP2.3* promoter repressed its expression and was strongly associated with taproot shape variation, revealing a TE-mediated cis-regulatory mechanism underlying morphological divergence. Functional analyses showed that *RsOFP2.3* overexpression (OE) promotes radial expansion by enhancing cambial cell division and xylem differentiation, resulting in thicker and shorter taproots, whereas silencing *RsOFP2.3* produced opposite phenotypic effects. Mechanistically, RsOFP2.3 physically interacts with the TONNEAU1-recruiting motif (TRM) protein RsTRM4, recruiting it from microtubules to the cytoplasm. Downregulation of *RsTRM4* reduced taproot length, while *RsTRM4* OE partially alleviated the shortened organ phenotypes caused by *RsOFP2.3* OE, indicating an antagonistic relationship that fine-tunes organ morphology. These findings uncover two coordinated regulatory mechanisms involving TE-mediated cis-regulatory variation and a conserved OFP-TRM interaction module that jointly shape the balance between radial and longitudinal growth during radish taproot development, providing valuable molecular targets for precision breeding of storage root crops.

## Introduction

Organ shape is a critical determinant of agronomic performance and commercial value, influencing appearance quality, harvest efficiency, postharvest processing, market classification, and consumer acceptance [1]. Considerable advances have been achieved in elucidating the molecular mechanisms underlying shape regulation in fleshy fruits, such as tomato, cucumber, peach, melon, apple, and pepper [2–9], as well as in cereals, where grain shape directly impacts yield and market value [10–14]. By contrast, our understanding of shape regulation in swollen storage organs, such as tubers and fleshy roots, remains limited, despite their agronomic and economic significance.

Radish (*Raphanus sativus* L.), a member of the *Brassicaceae*, is primarily cultivated as a root vegetable and exhibits remarkable diversity in taproot morphology. Although recent studies in potato, beet, carrot, and radish have identified candidate regulators of storage organ shape [15–20], knowledge in this area still lags far behind that of fruits and grains. Importantly, radish represents a unique case among storage crops: unlike potato tubers, which develop from stolons, or beet storage roots, which derive from root tissues, the fleshy taproot of radish develops from both the hypocotyl and a portion of the root, expanding through secondary growth and massive proliferation of xylem parenchyma [18, 21–23]. These distinctive structural and developmental features make radish a valuable model system for elucidating the genetic and developmental basis of storage organ shape, complementing insights gained from studies of fruits, grains, and other root crops.

Organ shape variation arises primarily from differences in cell division and expansion, processes tightly regulated by microtubule organization [24]. The preprophase band (PPB) plays a pivotal role in defining the division plane, ensuring proper spindle orientation [24]. Proper PPB assembly depends on the TON1/TRM/PP2A (TTP) complex, in which TONNEAU1-recruiting motif (TRM) proteins recruit TON1 to cortical microtubules [25, 26], thereby linking cytoskeletal organization to organ shape determination. In *Arabidopsis*, TRM2/LNG1 overexpression (OE) promotes extensive organ elongation, affecting leaves, petioles, floral organs, and siliques [27]. TRM homologs have also been identified in other plant species, where their functional roles in organ development have begun to be elucidated. For example, in cucumber, CsTRM5 was reported to regulate cucumber fruit shape *via* modulating the orientation of cell division and the extent of cell enlargement [28].

One major advance in understanding organ morphology has been the identification of OVATE family proteins (OFPs) and TRM proteins as key regulators of organ shape [7]. OFPs, unique to plants, are defined by the presence of an evolutionarily conserved OVATE domain [29], and have been shown to control organ shape in Arabidopsis as well as in diverse crops such as rice, tomato, potato, and cucumber [7, 17, 30, 31]. OFPs OE typically produces dwarfed plants, characterized by shorter and broader organs, including kidney-shaped cotyledons or more rounded fruits [32]. In tomato, OFPs physically interact with TRM proteins *via* their conserved M8 motif, leading to dynamic relocalization of both partners between the cytoplasm and microtubule arrays [7]. This interaction reshapes cell proliferation patterns along longitudinal and transverse directions to fine-tune fruit morphology. Genetic evidence further demonstrates that mutations occurring in *Sltrm3/4* and *Sltrm5* synergistically suppress the fruit elongation in the *ovate/Slofp20 (o/s)* background, restoring a more rounded shape. In contrast, loss-of-function in *Sltrm19* or *Sltrm17/20a* promotes further elongation, thereby intensifying the obovoid phenotype of the *o/s* background, underscoring the combinatorial nature of the OFP-TRM module in bilateral regulation of organ shape [8]. Whether this conserved module operates in radish taproots, a cambium-driven storage organ distinct from both fruit pericarps and roots, remains an open and intriguing question.

Genome-wide association studies (GWAS) provide a powerful approach to dissect the genetic architecture of complex traits in plants. Structural variants (SVs), including insertions and deletions in coding or regulatory regions, can strongly influence gene expression and phenotype [33]. Moreover, SV-based GWAS has been shown to improve the power of association mapping compared to single nucleotide polymorphism (SNP)-based approaches in some crops [34]. Transposable elements (TEs) are now recognized as important cis-regulatory drivers, capable of introducing novel transcription factor binding sites or repressing gene activity when inserted into promoter regions [35]. Such TE-derived insertions can modulate the expression of adjacent genes and contribute to morphological diversification. However, whether TE-derived promoter variation contributes to natural diversity in radish taproot shape has not been addressed.

In this study, we combined SV-GWAS with cellular and molecular analyses to uncover the genetic basis of radish taproot shape. We identified RsOFP2.3 as a key regulator of radish taproot morphology, where a TE-derived promoter insertion represses its expression and contributes to natural variation in root shape. Using complementary gain- and loss-of-function analyses in radish, we demonstrate that RsOFP2.3 plays an essential role in controlling taproot shape. Cellular analyses based on RsOFP2.3 OE further reveal that RsOFP2.3 promotes radial thickening by enhancing cambial activity and parenchyma cell proliferation. Moreover, RsOFP2.3 physically associates with RsTRM4, uncovering a conserved OFP-TRM regulatory module and a TE-derived regulatory mechanism contributing to storage organ morphogenesis.

## Results

### Structural variant**-**based genome-wide association studies identifies RsOFP2.3 as a key regulator of taproot shape

To elucidate the genetic basis of fleshy taproot morphology in radish, we performed whole-genome resequencing of 144 East-Asia big long radish accessions (*R. sativus* var. *hortensis*) (Table S1), generating high-quality paired-end reads with an average sequencing depth of 20×. Clean reads were aligned to a newly assembled XLM reference genome, enabling accurate detection of SVs across the population. A total of 25 686 high-confidence SVs were identified, including insertions, deletions, inversions, and duplications, each supported by stringent quality metrics (e.g. read depth, split read support, and breakpoint confidence). Using these SVs as markers, we conducted an SV-based GWAS with root shape index (length/width ratio) as the primary phenotype (Fig. S1). This analysis identified 50 SV loci significantly associated with variation in fleshy taproot shape [−log_10_(*P*) > 4.0, Bonferroni-corrected] (Table S2). Among them, a prominent association peak was detected on chromosome 2, overlapping with the gene *RsOFP2.3*, an OFP previously implicated in regulating taproot shape in our earlier study (Table S3 [19]). These results suggest that *RsOFP2.3* is a preferential candidate gene for controlling taproot shape.

### *RsOFP2.3* expression correlates with taproot morphology and is enriched in cambial tissues

**Figure 1:**
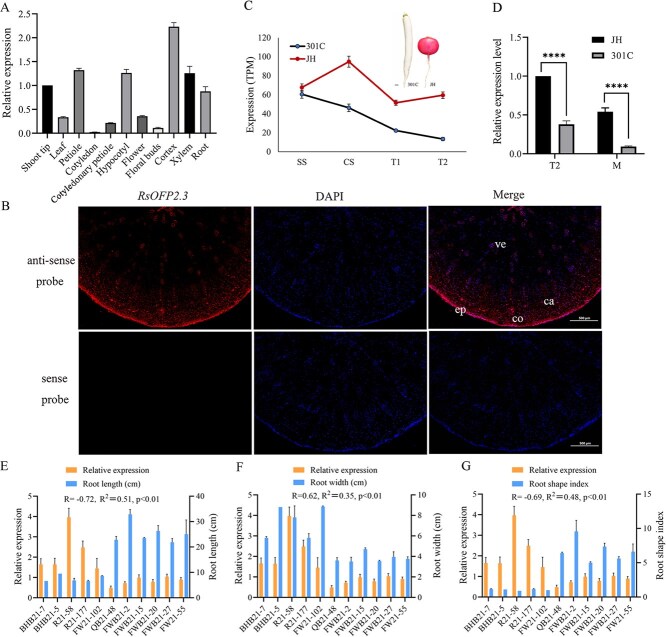
*RsOFP2.3* expression in radish. (A) *RsOFP2.3* relative expression level in different organs and tissues of radish. (B) RNA-FISH in taproot of round-rooted radish line JH. The upper panel shows the signal detected with the *RsOFP2.3* antisense probe, DAPI-stained root structure, and merged view, respectively. The lower panel shows no detectable signal with the sense probe of *RsOFP2.3.* Ep, epidermis; co, cortex; ca, cambium; ve, vessel. Scale bars, 500 μm. (C) *RsOFP2.3* expression in round-rooted radish JH and long-rooted radish 301C during four different development stages based on RNA-seq data [36]. Images of the two taproots are shown. Scale bar, 1 cm. SS, seedling stage; CS, cortex split stage; T1, thickening stage I; T2, thickening stage II. (D) Relative expression of *RsOFP2.3* in dissected taproot tissues of JH and 301C samples enriched in epidermal, cortex, and cambial cells at T2 and mature stage (M). ^****^P < 0.0001, as determined by Student’s *t*-test compared to JH at the respective stage. (E–G) *RsOFP2.3* expression and taproot length (E), width (F), root shape index (G) statistics in natural radish lines.

To further characterize the expression and potential function of *RsOFP2.3* in radish, we conducted a comprehensive expression analysis across multiple tissues and developmental stages. Tissue-specific expression profiling revealed that *RsOFP2.3* was broadly expressed in various organs/tissues, with relatively high expression observed in shoot tip, petiole, hypocotyl, cortex, xylem, and root. Notably, the highest expression was detected in cortex regions which includes epidermis, cortex, and cambium tissues (Fig. 1A). RNA fluorescence *in situ* hybridization (RNA-FISH) further showed that *RsOFP2.3* transcripts were localized mainly in the epidermis, cortex, cambium, and xylem vessels, consistent with its role in root thickening and expansion (Fig. 1B). Developmental stage-specific expression analysis in two contrasting radish genotypes using RNA-seq data, revealed distinct temporal expression patterns (Fig. 1C). In long-rooted accession 301C, *RsOFP2.3* expression gradually decreased as the taproot developed. In contrast, in the round-rooted accession JH, *RsOFP2.3* expression was consistently higher than in 301C at most stages, except at the seedling stage (SS). These differences suggest that *RsOFP2.3* may contribute to genotype-specific differences in root morphology. Quantitative real-time polymerase chain reaction (qRT-PCR) using dissected taproot tissues enriched in epidermal, cortex, and cambial cells further confirmed that *RsOFP2.3* expression was significantly higher in JH than in 301C, strengthening the correlation between high gene expression and round-root phenotype (Fig. 1D).

To further explore the association between *RsOFP2.3* expression and taproot morphology, we quantified *RsOFP2.3* transcript levels across 11 inbred radish lines exhibiting diverse taproot shapes. *RsOFP2.3* expression showed a strong negative association with root length (*R* = −0.72, *R*^2^ = 0.51, *P* < 0.01) and root shape index (*R* = −0.69, *R*^2^ = 0.48, *P* < 0.01), while exhibiting a positive relationship with root width (*R* = 0.62, *R*^2^ = 0.35, *P* < 0.01) (Fig. 1E−G). These results showed that *RsOFP2.3* expression levels account for approximately 35%–50% of the observed morphological variation, suggesting that *RsOFP2.3* plays a major role in determining the balance between radial thickening and longitudinal growth of the taproot.

### A transposable element**-**derived promoter insertion represses *RsOFP2.3* expression and contributes to taproot shape variation

**Figure 2:**
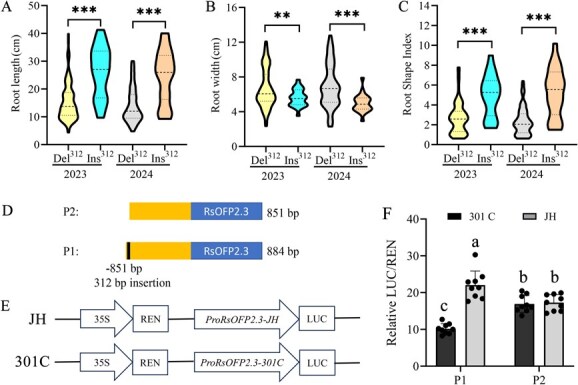
Natural variations of *RsOFP2.3* in radish. (A–C) Analysis of *RsOFP2.3* promoter indel using phenotypic data from 2 years. ^**^P < 0.01, ^***^P < 0.001, Student’s *t*-test. (D) Different fragments of the *RsOFP2.3* promoter and their schematic representations. P1: 884 bp upstream of the ATG start codon; P2: 851 bp upstream of the ATG start codon; position of the 312 bp insertion was indicated. (E) Diagrams of the reporter vectors used in the dual-LUC assay. 301C and JH constructs harbor the firefly luciferase reporter under the control of *RsOFP2.3* promoters from the corresponding lines. (F) Transient LUC expression driven by P1 and P2 promoter fragments of 301C and JH in Arabidopsis protoplasts. Values are shown as mean ± SE (*n* = 9, independent biological replicates). Distinct letters denote statistically significant differences identified by two-way analysis of variance (ANOVA) followed by Tukey’s HSD test (*P* < 0.05).

To investigate sequence variation in *RsOFP2.3*, the full-length gene together with approximately 2000 bp of the upstream promoter region relative to the ATG start codon was cloned from two contrasting radish genotypes: JH and 301C. Sequence comparison revealed a 312-bp insertion in the promoter of 301C that was absent in JH (Fig. S2). Population-level association analysis demonstrated that this insertion is significantly correlated with taproot morphology, being associated with increased root length, reduced root width, and a higher root shape index (Fig. 2A–C). Repeat annotation using CENSOR (https://www.girinst.org/censor/index.php) indicated that this sequence corresponds to a truncated fragment of L1-94_RSa, a LINE-type non-LTR retrotransposon from the *R. sativus* genome, with 75.3% sequence similarity over a 294-bp region ([37]; Fig. S3). The insertion is flanked by identical 6-bp direct repeats (CAAAAC), characteristic of target site duplications generated upon TE integration, confirming its transposon origin. Whole-genome BLASTn analysis identified additional loci with high similarity to the 312-bp sequence, suggesting that it belongs to a multicopy TE family in radish (Fig. S4). Dual-luciferase (LUC) reporter assays revealed that, while other minor promoter variants had little effect, the 312-bp insertion significantly suppressed promoter activity (Figs S2 and 2D–F). Collectively, these findings indicate that the 312-bp promoter insertion represents a TE-derived cis-regulatory element that represses *RsOFP2.3* expression, thereby contributing to natural variation in radish taproot shape.

### RsOFP2.3 regulates fleshy taproot morphology in radish

**Figure 3:**
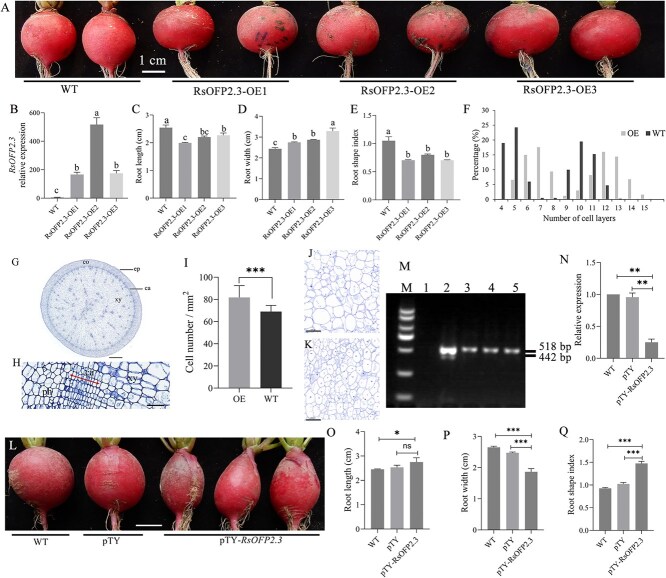
RsOFP2.3 positively regulates fleshy taproot width in radish. (A) Representative taproots from WT and *RsOFP2.3*-OE plants at mature stage. Scale bar, 1 cm. (B) RT-qPCR analysis of *RsOFP2.3*-OE transgenic plants. *RPII* was used as the endogenous control. (C–E) Taproot length (C), width (D) and taproot shape index (E) of WT and *RsOFP2.3*-OE plants at mature stage. Values are shown as mean ± SE (*n* = 9). Distinct letters denote statistically significant differences according to one-way ANOVA followed by Tukey’s HSD test (*P* < 0.05). (F) Quantification of cambium cell layers in WT and *RsOFP2.3*-OE taproots (*n* = 500). (G) Representative cross section showing the general anatomical organization of a radish taproot. co, cortex; xy, xylem; ca, cambium; ep, epidermis. Scale bar = 1000 μm. (H) Enlarged view of a typical cambial region. ph, phloem; xy, xylem; ca, cambium. Scale bar = 50 μm. (I) Quantification of cell number per unit area (1 mm^2^) in WT and *RsOFP2.3*-OE taproots. (J and K) Cross sections highlighting xylem parenchyma cells in WT (J) and *RsOFP2.3*-OE (K) taproots. Scale bar = 200 μm. (L–Q) Virus-induced silencing of *RsOFP2.3* in radish. Phenotypes (L), PCR amplification using pTY-F/pTY-R primer, M, marker 2000; lane 1, WT; lane 2, pTY; lanes 3–5, pTY-RsOFP2.3 (M), *RsOFP2.3* relative expression level (N), root length (O), root width (P) and root shape index (Q) in control plant, pTY empty vector plant and *RsOFP2.3* silenced plants. Scale bar = 1 cm. Values are shown as mean ± SE. Statistical significance was determined using Student’s *t*-test (^***^P < 0.001, ^**^P < 0.01, ^*^P < 0.05).

To validate the functional role of *RsOFP2.3* in regulating fleshy taproot morphology, we generated transgenic radish plants overexpressing *RsOFP2.3* (OE lines) driven by the CaMV 35S promoter (Fig. 3A). The integration of the transgene was validated through genomic PCR using a forward primer in the CaMV 35S promoter and a reverse primer in the *RsOFP2.3* coding region, followed by RT-qPCR to quantify transgene expression levels (Figs S5 and 3B). Assessment of phenotype in transgenic plants revealed that OE of *RsOFP2.3* led to a significant increase in root width and a concomitant reduction in taproot length, resulting in a substantial decrease in root shape index (Fig. 3C–E). These findings provide direct evidence that *RsOFP2.3* promotes radial expansion while repressing longitudinal growth of the fleshy taproot.

To investigate the cellular basis underlying the altered taproot morphology in *RsOFP2.3*-OE lines, we examined transverse sections of mature taproots and quantified the number of cambial cell layers across different regions of root cross-sections and analyzed their distribution patterns. Transverse sections of taproots showed a significant increase in vascular cambial cell layer number in *RsOFP2.3*-OE lines (Fig. S6). Moreover, as shown in Fig. 3F, the distribution of cambial cell layer numbers differed markedly between OE and wild-type (WT) plants. In WT taproots, the majority of radial files exhibited 4–5 and 10–11 cambial cell layers, with a peak at 5 layers (~25%). In contrast, OE lines showed a broader distribution skewed toward higher layer numbers, with a notable increase in radial file exhibiting 6–7 and 12–13 layers. Quantitatively, OE plants had a significantly higher proportion of cells with ≥12 layers, while the proportion of 4–5 layers was reduced compared to WT. In addition to the increased number of cambial layers, OE taproots displayed a higher cell density within a defined unit area compared to WT (Fig. 3G–K). These results indicate that RsOFP2.3 OE promotes cambial activity and cell proliferation, leading to an increased number of cells per unit area. Although cell expansion is reduced in the OE lines, as evidenced by smaller cell size compared with the WT, the increase in cell number predominates, ultimately resulting in radial thickening and increased taproot width.

To further validate the role of RsOFP2.3 in regulating taproot morphology in radish, virus-induced gene silencing (VIGS) approach was employed to transiently reduce *RsOFP2.3* expression in planta (Fig. 3L–Q). qRT-PCR analysis further showed that the transcript level of *RsOFP2.3* was markedly reduced in pTY-RsOFP2.3 infected plants compared with plants treated with the empty vector control (pTY-S) or mock-treated plants (Fig. 3N), indicating effective gene silencing. Phenotypic analysis revealed that VIGS-mediated knockdown of *RsOFP2.3* resulted in pronounced changes in taproot morphology. Compared with control plants, *RsOFP2.3*-silenced plants exhibited a significant decrease in taproot width, accompanied by an increase in root length, leading to a higher root shape index (Fig. 3O–Q). These phenotypic alterations were opposite in direction to those observed in *RsOFP2.3*-OE lines, supporting a positive role of *RsOFP2.3* in promoting radial expansion while restricting longitudinal growth of the fleshy taproot. Taken together, the VIGS results provide independent loss-of-function evidence that *RsOFP2.3* is required for proper regulation of taproot shape in radish, complementing the gain-of-function phenotypes observed in stable OE lines.

### RsOFP2.3 physically interacts with RsTRM4 and alters its subcellular localization

**Figure 4:**
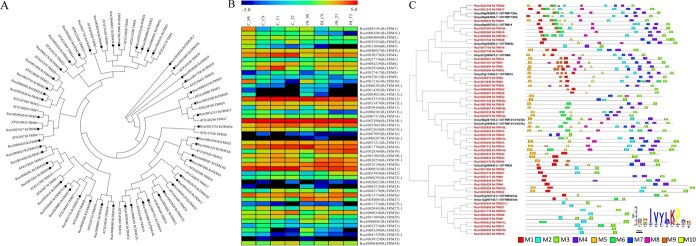
*RsTRMs* in radish. (A) Phylogenetic analysis of TRM family proteins in radish. The phylogenetic tree displays only the topology. Maximum likelihood tree constructed from full-length amino acid sequences of 50 RsTRM proteins identified in the radish genome and 34 Arabidopsis TRMs. The tree was generated using ClustalX2 (version 2.0.12) with 1000 bootstrap replicates. TRMs in radish were indicated by diamond symbols. (B) Expression profiling of *RsTRM* genes in fleshy taproot tissues of 301C and JH radish at four different developmental stages. A heat map showing the expression patterns of 50 *RsTRM* genes detected in fleshy taproot tissues based on RNA-seq data [36]. Gene expression levels were normalized as log_2_ (TPM). The scale bar indicates relative expression levels. (C) Phylogenetic analysis and conserved motifs distribution of RsTRMs and SlTRMs. The conserved motifs are shown as distinct motif types. The M10 motif, which shows high sequence similarity to the M8 motif of tomato TRM proteins, was identified and shown.

**Figure 5:**
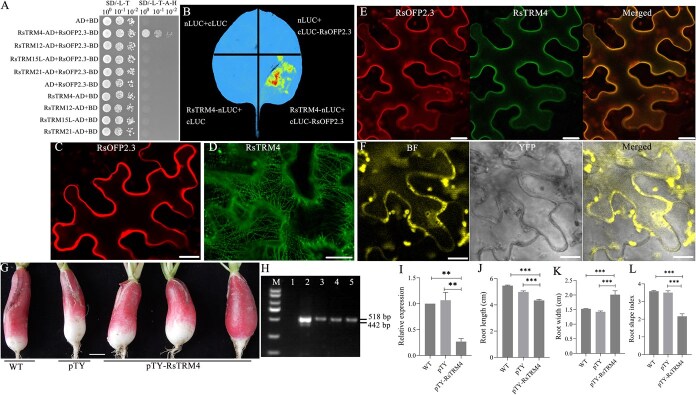
RsOFP2.3 physically interacts with RsTRM4 and alters its subcellular localization. (A) Interaction of RsOFP2.3 with RsTRM4, RsTRM12, RsTRM15L, and RsTRM21 detected by a Y2H assay. The co-transformation of empty prey (pGADT7) and bait (pGBKT7) vectors was used as the negative control. Coding sequences of *RsOFP2.3* was cloned into the pGBKT7 plasmid, and *RsTRM4*, *RsTRM12*, *RsTRM15L*, and *RsTRM21* were cloned into the pGADT7 plasmid. Yeast cells carrying both constructs were serially diluted (1×, 10×, and 100×) and spotted onto SD/-T-L plates to confirm co-transformation. Growth on SD/-T-L-H-A medium indicated positive interactions. SD, minimal medium; T, tryptophan; L, leucine; H, histidine; and A, adenine. (B) LUC complementation imaging (split-LUC) assays confirming the physical interaction between RsOFP2.3 and RsTRM4 *in planta*. (C and D) Subcellular localization of RsOFP2.3 (C) and RsTRM4 (D) expressed individually in *N. benthamiana* leaf epidermal cells. (E) Co-expression of RsOFP2.3 and RsTRM4 in *N. benthamiana* epidermal cells. (F) Confirmation of the interaction between RsOFP2.3-cYFP and nYFP-RsTRM4 in epidermal cells of *N. benthamiana* leaves using BiFC assay. nYFP and cYFP represent the amino- and carboxyl-terminal fragments of YFP, respectively. Scale bar, 20 μm. (G–L) Virus-induced silencing of *RsTRM4* in radish. Phenotypes (G), PCR amplification using pTY-F/pTY-R primer, M, marker; lane 1, WT; lane 2, pTY; lanes 3–5, pTY-RsTRM4 (H), *RsTRM4* expression level (I), root length (J), root width (K) and root shape index (L) in control plant, pTY empty vector plant and RsTRM4 silenced plants. Scale bar, 1 cm. Values are shown as mean ± SE. Statistical significance was determined using Student’s *t*-test (^***^P < 0.001, ^**^P < 0.01).

Findings from prior investigations using tomato and Arabidopsis as model systems have demonstrated that OFPs regulate organ morphology through physical interactions with TRM proteins. To explore whether a similar conserved regulatory module operates in radish, we performed a genome-wide search and identified 50 *RsTRM* genes, which were named based on their homology with Arabidopsis TRM family members. Subsequent transcriptome profiling further revealed that 39 of these genes are expressed in fleshy taproot tissues (Fig. 4A and B). Given that TRM-OFP interactions are known to depend on the conserved M8 motif within TRM proteins. We next conducted motif analysis, which revealed that 19 of the 50 RsTRMs proteins harbor M8/M10 motif (in this study) (Fig. 4C). Among these candidates, RsTRM4, RsTRM12, RsTRM15L, and RsTRM21 exhibited differential expression between round-rooted and long-rooted accessions, thereby suggesting their potential involvement in taproot shape determination. Subsequently, yeast two-hybrid (Y2H) assays revealed that only RsTRM4 exhibited a direct interaction with RsOFP2.3 (Fig. 5A). To further substantiate this finding, the interaction was validated using split-LUC complementation assays, which confirmed the physical association between the two proteins in planta (Fig. 5B). To further explore the subcellular behavior of these proteins and the potential effect of their interaction on localization, we transiently expressed each protein individually or in combination in *Nicotiana benthamiana* leaf epidermal cells. RsOFP2.3 mainly localized to the cytoplasm, which was further supported by its colocalization with the cytoplasmic/nuclear localization reference CsOVATE-GFP (Figs 5C and S7A). In contrast, RsTRM4 was predominantly associated with the cytoskeleton and colocalized with the microtubule marker MBD-mCherry (Figs 5D and S7B). However, upon co-infiltration, RsTRM4 was relocalized from the cytoskeleton to the cytoplasm, where it colocalized with RsOFP2.3 (Fig. 5E), suggesting a recruitment effect mediated by the interaction. This observation was further supported by bimolecular fluorescence complementation (BiFC) assays, which confirmed that RsOFP2.3 and RsTRM4 physically interact and colocalize in the cytoplasm (Figs 5F and S8). The more restricted BiFC signal reflects localized sites of RsOFP2.3–RsTRM4 interaction within the cytoplasm, whereas co-expression assays show the overall redistribution of RsTRM4 from the cytoskeleton to the cytoplasm.

Given the physical interaction between RsTRM4 and RsOFP2.3 and the relocalization of RsTRM4 upon co-expression, we next examined whether RsTRM4 is functionally involved in taproot shape regulation in radish using a VIGS approach (Fig. 5G–L). Phenotypic analysis revealed that RsTRM4 silencing resulted in clear alterations in taproot morphology. Compared with control plants, pTY-RsTRM4 infected plants displayed reduced taproot length together with increased root width, leading to a significantly lower root shape index (Fig. 5I–L). These phenotypic changes indicate enhanced lateral growth and compromised longitudinal elongation of the fleshy taproot upon reduction of *RsTRM4* expression.

### RsTRM4 antagonizes RsOFP2.3 function to balance lateral and longitudinal growth

**Figure 6:**
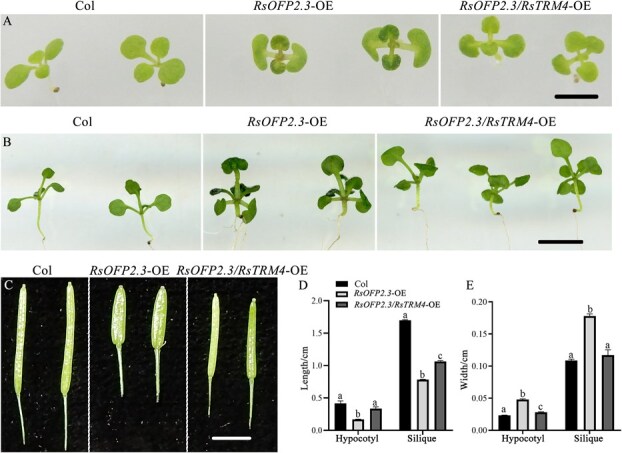
*RsTRM4* OE partially rescues the organ shape defects caused by *RsOFP2.3* OE. (A) Cotyledon morphology of wild-type (WT), *RsOFP2.3* OE, and *RsOFP2.3/RsTRM4* co-OE (*RsOFP2.3/RsTRM4*-OE) seedlings. RsOFP2.3-OE lines exhibit kidney-shaped cotyledons, which are partially normalized in OE/OE lines. (B) Hypocotyl morphology of the three genotypes. *RsOFP2.3* OE leads to shorter and thicker hypocotyls, while OE/OE lines display partial recovery of elongation. (C) Silique morphology of WT, RsOFP2.3-OE, and *RsOFP2.3/RsTRM4*-OE plants. RsOFP2.3-OE siliques are shortened and thickened, whereas *RsOFP2.3/RsTRM4*-OE siliques show increased length and reduced width. (D and E) Quantification of hypocotyl and silique lengths (D) and widths (E) in WT, RsOFP2.3-OE, and *RsOFP2.3/RsTRM4*-OE plants. Each value represents the mean ± SE of nine replicates. Significant differences among genotypes were determined by Tukey’s test (*P* < 0.05) and are indicated by different letters above the bars. Scale bars, 1 cm (A–C).

To investigate the potential genetic interaction of RsTRM4 with RsOFP2.3, we overexpressed *RsTRM4* in a stable *RsOFP2.3*-overexpressing *Arabidopsis thaliana* line to generate double OE plants (*RsOFP2.3/RsTRM4*-OE). As previously observed, *RsOFP2.3*-OE plants exhibited pleiotropic organ shape alterations, including kidney-shaped cotyledons, shortened and thickened hypocotyls, and swollen siliques (Fig. 6A–C; [19]). In contrast, the *RsOFP2.3/RsTRM4*-OE lines displayed a partial restoration of these phenotypes (Fig. 6A–C). Cotyledon morphology appeared more normalized, and both hypocotyl and silique elongation were visibly improved compared to *RsOFP2.3*-OE. Quantitative measurements confirmed these observations: hypocotyl and silique lengths were significantly increased, while their widths were reduced in *RsOFP2.3/RsTRM4*-OE lines relative to *RsOFP2.3*-OE, although silique length and hypocotyl width were not fully restored to WT levels (Fig. 6D and E). These results suggest that RsTRM4 promotes longitudinal growth and partially antagonizes the enhanced lateral expansion induced by *RsOFP2.3* OE. Together, these findings indicate that RsTRM4 genetically interacts with RsOFP2.3, likely functioning as a buffering factor to fine-tune organ shape by balancing lateral and longitudinal expansion during organ development.

## Discussion

Radish is cultivated worldwide primarily for its enlarged fleshy taproot, whose shape strongly influences transport efficiency, processing suitability, and consumer preference, making it a key breeding target. Understanding the molecular mechanisms governing storage root morphology will not only accelerate radish improvement but also broaden our knowledge of root organogenesis. In this study, we identified RsOFP2.3 as a major regulator of taproot shape, supported by direct evidence from stable transgenic lines and also the VIGS lines, and demonstrated that a TE-derived promoter insertion contributes to natural variation in taproot shape. Furthermore, we revealed that the RsOFP2.3-RsTRM4 module fine-tunes taproot morphology. This study highlights both a cis-regulatory mechanism and a conserved protein interaction module underlying shape diversification in radish.

### RsOFP2.3 modulates radish taproot morphology by altering cambial cell patterning

OFPs, named after the *ovate* locus responsible for pear-shaped tomato fruit, are a plant-specific multigene family characterized by a conserved OVATE domain [11, 38]. They play critical roles in diverse aspects of plant growth and development, particularly in determining organ morphology. The functional consequences of OFPs can arise from either sequence variation or altered expression levels. In tomato, an SNP in OVATE introduces an early stop codon, leading to a truncated protein that converts fruit shape from round to elongated [38]. In pepper, fruit shape differences between the cultivars ‘Mytilini Round’ and ‘Piperaki Long’ are attributed to distinct *CaOvate* expression patterns rather than mutations in its ORF. Higher *CaOvate* expression was detected in ‘Mytilini Round’, and its downregulation by VIGS, leads to a shift toward oblong fruit morphology [6]. Similarly, in *Citrus*, *CitOFP19* showed significantly higher expression in round pummelo ovaries compared with pear-shaped ones [39]. Collectively, these findings suggest that *OFP* expression level is closely associated with organ shape regulation across species. Consistent with this pattern, our study revealed that *RsOFP2.3* was highly expressed in the round taproot radish cultivar JH and its expression was significantly associated with taproot shape variation at population level (Fig. 1). OE of *RsOFP2.3* increased taproot width while reducing taproot length, resulting in an oblate taproot with a decreased shape index, while it was opposite in the silencing lines (Fig. 3). This phenotype aligns with the general function of OFPs in other plants, where their OE often leads to shorter and thicker organs [32].

Mechanistically, OFPs modulate organ shape through diverse cellular processes that vary across species, organs, and developmental contexts. In Arabidopsis, AtOFP1 reduced aerial organ length by decreasing cell elongation [40]. In tomato, OVATE and SlOFP20 primarily affected fruit elongation mainly through changing the cell division pattern at the proximal end of fruits [7]. Similarly, CaOFP20 regulates fruit morphology by impacting cell division in pepper [41]. In contrast, in radish, *RsOFP2.3* OE regulates taproot shape through a distinct mechanism, primarily by enhancing cambial activity. This leads to the differentiation of more xylem parenchyma cells, ultimately driving radial thickening of the taproot (Fig. 3). Notably, this effect is consistent with *RsOFP2.3*’s expression in the vascular cambium. This divergence underscores how the function of OFPs can be adapted to the specific developmental needs of different organs. Evidence from other species, such as OsOFP2 in rice, supports the role of OFPs in vascular development, as its OE leads to altered vascular bundle positioning [42]. Therefore, although RsOFP2.3 and OVATE/SlOFP20 share some functional similarities, particularly in their involvement in cell division regulation, their distinct roles in organ development-root versus fruit, highlight the specialized functions of OFPs across plant species and organs. This distinction expands our understanding of OFP proteins’ conserved yet diversified roles, from aerial organ development in fruits to underground organ development in roots, and provides insight into their unique contributions to plant morphology.

### A transposable element-derived promoter insertion in *RsOFP2.3* contributes to taproot shape variation in radish

Genomic structural variations, such as deletions, insertions, inversions, and segmental duplications, represent a major source of genomic diversity and have profound effects on phenotypic variation [43]. Among them, TE insertions are increasingly recognized as key drivers of regulatory evolution and phenotypic diversification. TEs can modulate gene expression through various mechanisms, such as introducing regulatory motifs that alter the transcriptional activity of nearby genes [44]. Notably, several organ-shape loci across species have been linked to SVs affecting OFP expression, thereby influencing organ morphology. In peach, a heterozygous structural inversion of about 1.67 Mb enhances *PpOFP2* expression, resulting in the development of flat-shaped fruits [3], whereas in pepper, a 42-bp deletion located upstream of *CaOFP20* shows a strong correlation with fruit shape diversity [45]. Likewise, the Rider retrotransposon insertion upstream of SUN in tomato triggers ectopic expression and produces elongated fruit [46].

In this study, we identified a 312-bp TE-derived insertion in the *RsOFP2.3* promoter that represses its expression and is strongly associated with taproot shape variation in radish. Accessions carrying the insertion exhibited elongated taproots, whereas those lacking it developed round roots with increased radial thickening. Whole-genome BLASTn analysis revealed that the 312-bp sequence belongs to a multicopy TE family, suggesting that similar insertion events may have occurred repeatedly during radish genome evolution. This TE insertion thus represents a cis-regulatory variant that modulates the expression of a key developmental regulator without altering its protein-coding sequence, providing direct evidence that TE activity can generate heritable phenotypic diversity in root morphology.

The presence of a multicopy TE family and its association with root-shape traits across natural populations suggest that TE-mediated regulatory changes may have been exploited during radish domestication and diversification. Such promoter insertions offer a flexible and efficient mechanism for morphological innovation, enabling rapid shifts in developmental gene expression under selective pressures. Similar to the domestication-related *tb1* enhancer TE insertion in maize [47], the *RsOFP2.3* TE insertion may have facilitated the emergence of diverse taproot architectures adapted to distinct ecological conditions and consumer preferences. Collectively, these findings highlight how TE-driven cis-regulatory evolution, together with the conserved OFP-TRM module, jointly shape organ morphology and contribute to the diversification of storage organ traits in root crops.

### RsOFP2.3-RsTRM4 constitutes a conserved and complex module regulating taproot shape morphogenesis

Microtubule-associated proteins are known to interact, both genetically and physically, with key determinants of fruit shape [48]. The OFP-TRM module provides a well-studied example. In this system, OFPs interact with TRMs *via* the M8 motif, which affects the intracellular distribution of the protein complex. These changes subsequently alter microtubule organization and cell division orientation, influencing the final fruit shape. In Arabidopsis, transgenic plants overexpressing *AtTRM1* and *AtTRM2* (LONGIFOLIA2 and LONGIFOLIA1, respectively) results in organ elongation, whereas the *trm1trm2* double mutant exhibits reduced organ length due to decreased cell elongation [27]. Both proteins contain the conserved M8 motif, suggesting that their function depends on interactions with OFPs [32]. Similarly, in maize, ZmLNG1—a homolog of Arabidopsis TRMs, acts as a molecular bridge connecting ZmOFPs to the TTP complex, which likely modulates ZmOFP phosphorylation and thereby influences organ morphology [30]. In tomato, mutations in *SlTRM3/4* and *SlTRM5* act additively to revert the elongated fruit phenotype of the *ovate/Slofp20* (*o/s*) mutant to a round shape, whereas mutations in *SlTRM19* and *SlTRM17/20a* promote fruit elongation and further enhance the obovoid phenotype of *o/s*. Notably, the additive effects of SlTRM3/4 and SlTRM5 are evident mainly in the *o/s* background rather than in the WT [8].

Consistent with observations in other species, we found that silencing *RsTRM4* resulted in reduced taproot length, whereas OE of *RsTRM4* in Arabidopsis partially antagonized the phenotypic effects caused by *RsOFP2.3* OE. However, in the WT (Col) background, *RsTRM4*-overexpressing plants showed only subtle morphological differences compared with the control (Figs S9 and S10), and the effect was much weaker than that observed in the *RsOFP2.3*-OE background. Similar buffering interactions have also been observed in tomato, where different TRM alleles either suppress or enhance OFP-mediated fruit elongation [8]. Overall, the OFP-TRM module constitutes a conserved yet functionally diversified mechanism controlling organ shape across species. Different TRM members may either counteract or reinforce OFP activity, indicating a complex regulatory network that fine-tunes organ morphology and contributes to developmental robustness. Furthermore, how *RsOFP2.3* OE leads to taproot shortening remains to be elucidated and will require further investigation through cytological analyses, identification of more RsOFP2.3-interacting proteins, and characterization of its downstream target genes.

## Conclusion

**Figure 7:**
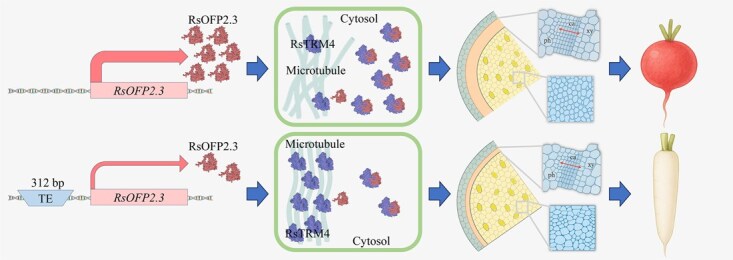
A working model of RsOFP2.3 in the regulation of radish taproot shape. When *RsOFP2.3* is highly expressed (upper panel), cytoplasmic RsOFP2.3 interacts with RsTRM4 and recruits it from the cytoskeleton to the cytoplasm, thereby altering RsTRM4 subcellular localization and likely its function which increases the number of cambial cell layers and xylem parenchyma cells, and promotes radial thickening of the taproot, resulting in a round-root phenotype. In contrast, when a 312-bp TE is inserted in the *RsOFP2.3* promoter (lower panel), *RsOFP2.3* expression is repressed, leaving most RsTRM4 associated with the cytoskeleton, which leads to fewer cambial layers and xylem parenchyma cells, thereby restricting radial expansion and producing a long-root phenotype.

This study establishes a mechanistic framework linking regulatory variation and protein interaction to control taproot shape in radish. RsOFP2.3 promotes radial thickening of the taproot by altering cambial cell patterning and stimulating parenchyma cell proliferation. A TE-derived promoter insertion reduces *RsOFP2.3* expression, thereby shifting growth toward longitudinal elongation and contributing to natural variation in root morphology. At the protein level, RsOFP2.3 physically interacts with RsTRM4, a TRM protein, and their co-localization pattern indicates that this interaction modulates cellular growth orientation. OE analyses further revealed that RsTRM4 partially counteracts RsOFP2.3-induced radial expansion, indicating an antagonistic yet coordinated relationship that fine-tunes organ morphology. Together, these results demonstrate how transcriptional regulation and protein interaction networks converge to balance radial and longitudinal growth. The proposed model (Fig. 7) illustrates how regulatory evolution and conserved OFP-TRM module jointly shape storage organ morphology, providing insights for the molecular design and breeding of root crops.

## Materials and methods

### Plant materials and growth conditions

A total of 144 East Asian big long radish (*R. sativus* L.) accessions were used for resequencing and GWAS. All accessions were cultivated under standard field conditions in fall of 2023 and 2024 at Tongzhou Experimental Station of the Beijing Vegetable Research Center, China. Taproot-related traits were measured at the mature stage. For transgenic validation, the inbred line RD02 was used for genetic transformation. Transgenic lines overexpressing *RsOFP2.3* and *RsTRM4* were generated and grown in controlled growth chambers under 16-hour light/8-hour dark photoperiod at 22°C.

### Structural variant-based genome-wide association study analysis

Isolation of genomic DNA from young leaf samples was performed using the DNAsecure Plant Kit (TIANGEN, China). Library preparation for paired-end sequencing (insert size around 350 bp) was performed, followed by sequencing on the DNBSEQ-T7 platform, generating approximately 4.5 Gb of clean data per accession. To construct a graph-based genome, the newly assembled *R. sativus* ‘XLM’ genome (RsXLM) was used as the reference, and construction of the variation graph was carried out using the vg software suite (v1.49.0) with default parameters [49]. SV genotyping was carried out by mapping Illumina short-read data from each accession to the graph-based genome through the vg map function, followed by quality-based filtering of SV calls.

Taproot length, maximum width, and root shape index (length/width) were measured and recorded for all 144 accessions at the harvest stage. SV-based GWAS was conducted using the GAPIT package (v3.0) in R (v4.4.0), implementing the Bayesian-information and Linkage-disequilibrium Iteratively Nested Keyway model to account for population structure and kinship [50]. A significance threshold of −log10(*P*) = 4.0 was applied to identify SVs significantly associated with target traits. These significant SVs were used to define candidate regions, and genes located within ±50 kb of each associated SV were considered as putative candidates. Manhattan and Q–Q plots were generated using the CMplot package (v4.3) in R.

### Gene expression analysis

Total RNA was extracted from various tissues and developmental stages using the RNAiso Plus RNA extraction kit (TaKaRa, Dalian, China). Total RNA was reverse-transcribed into first-strand cDNA using the Script™ RT Reagent Kit with gDNA Eraser (Perfect Real Time; TaKaRa, Dalian, China). qRT-PCR was then carried out employing TB Green® Premix Ex Taq™ (Tli RNaseH Plus; TaKaRa, Dalian, China). on a LightCycler480 system from Roche, Switzerland. Gene-specific primers are listed in Table S4. Expression normalization was carried out using RPII as the reference gene, and relative expression was quantified by the 2^–ΔΔCt^ method.

### RNA fluorescence *in situ* hybridization

To investigate the spatial expression pattern of *RsOFP2.3*, RNA-FISH was performed on paraffin-embedded radish root tissues using Cy3-labeled probes (Servicebio, Cat# GB21303). Fresh tissues about 0.5 cm thick from the middle part of JH taproot were rinsed in phosphate-buffered saline (PBS) and put in *in situ* hybridization fixative at 4°C for at least 12 hours. Dehydration was performed using a graded ethanol series, after which the samples were cleared in xylene and infiltrated with paraffin. Four-micrometer sections were mounted onto adhesive slides and dried at 62°C for 2 hours. Dewaxing and rehydration were carried out through a standard ethanol series followed by DEPC-treated water. After antigen retrieval and proteinase K digestion (5 μg/ml at 40°C), prehybridization was performed at 40°C for 1 hour. Cy3-labeled antisense probes were then applied, and hybridization step was conducted overnight at 40°C in a humid chamber. Post-hybridization washing was conducted using a series of saline-sodium citrate (SSC) with stepwise reduction in salt concentration. Nuclei were counterstained with 4′,6-diamidino-2-phenylindole (DAPI), and slides mounting was performed using an antifade reagent. Images were acquired using an upright fluorescence microscope (ECLIPSE Ci, Nikon).

### Transgenic vector construction and plant transformation

The RsOFP2.3 coding sequence, excluding the stop codon, was amplified using gene-specific primers and cloned into the KpnI- and BamHI-digested pCAMBIA2300 vector. The final construct placed RsOFP2.3 under the control of the CaMV 35S promoter and fused it with GFP at the C-terminal end. The resulting construct was confirmed by sequencing and introduced into *Agrobacterium tumefaciens* strain GV3101 and subsequently used to infect the inbred line of European radish (RDO2). The procedure for genetic transformation followed the method outlined by Xin et al. [51], in which cotyledonary petiole explants from young radish seedlings were used for *Agrobacterium*-mediated transformation. Briefly, explants were inoculated with *A. tumefaciens*, co-cultivated, and subsequently subjected to antibiotic selection and shoot regeneration, followed by rooting and acclimatization before transfer to soil. Positive transgenic plants were initially identified by genomic PCR using gene-specific primers (Table S4), and the OE of *RsOFP2.3* was further confirmed by RT-qPCR.

The CDS of *RsTRM4* without the stop codon was similarly cloned into pCAMBIA2300 between the KpnI and BamHI sites, driven by the CaMV 35S promoter and fused to a GFP tag at the 3′end. The verified construct was transformed into *Agrobacterium* strain GV3101 and then introduced into homozygous *RsOFP2.3*-OE *Arabidopsis* lines according to the floral dip transformation protocol [52]. Screening of positive transformants was first conducted on selective agar plates containing both hygromycin (20 mg/l) and kanamycin (50 mg/l) and subsequently validated by qPCR. Information on the primers employed for vector assembly is presented in Table S4.

### Virus-induced gene silencing assays

To further investigate the roles of *RsOFP2.3* and *RsTRM4* in regulating taproot shape in radish, a turnip yellow mosaic virus-based VIGS system was employed following a previously established strategy [53], with modifications adapted to the present study. For each target gene, a 36-nt exon-specific fragment was selected, and a corresponding 76-nt insert was designed. This insert comprised a 4-nt CTAG overhang at the 5′ end and a 72-nt palindromic sequence (consisting of the 36-nt specific fragment and its complementary strand). The insert was then synthesized and cloned into the SnaBI-linearized pTY vector *via* the CloneEZ cloning strategy. The resulting constructs, pTY-RsOFP2.3, pTY-RsTRM4, and the empty vector control pTY-S, were transformed into *Escherichia coli* Stb13 for large-scale plasmid amplification and purified using the OMEGA Plasmid Giga Kit (D6920). Purified plasmids were adjusted to a working concentration of 300–500 ng/μl prior to inoculation. For VIGS inoculation, two fully expanded true leaves of 2-week-old European small radish seedlings were used for each treatment. The pTY-RsOFP2.3 construct was inoculated into the round-rooted inbred line RDO2, in which *RsOFP2.3* shows relatively high endogenous expression, whereas pTY-RsTRM4 was inoculated into the long-rooted inbred line P41, in which *RsTRM4* is more highly expressed. Following inoculation, seedlings were maintained under low-light conditions at 22°C for 24 hours and subsequently transferred to a growth chamber at 22°C under a 16-hour light/8-hour dark photoperiod. A second inoculation was performed 5 days after the initial treatment, and a total of three to four inoculations were applied to ensure effective gene silencing. Seedlings inoculated with the empty pTY-S vector or treated with ddH_2_O were used as positive and negative controls, respectively. Taproot phenotypes were evaluated 1 week after the final inoculation. The silent lines were confirmed using virus-specific primers (Table S4), and the transcript levels of *RsOFP2.3* and *RsTRM4* were subsequently examined to assess gene silencing efficiency.

### Histological analysis

The middle portion of mature taproots was fixed in FAA solution (ethanol:acetic acid:formaldehyde, 45:6:5, v/v/v), embedded in paraffin, sectioned at 3–4 μm thickness, and dewaxed as previously described [36]. Toluidine Blue (Servicebio, G1032) staining was performed on tissue sections, which were subsequently imaged with a Nikon Eclipse E100 optical microscope fitted with a DS-U3 imaging unit. Cell numbers were quantified from cross sections by counting cells in 10 randomly selected fields (0.5 mm^2^ per field) using CaseViewer software (v2.4.0). Cambial cell layer numbers were determined by examining 100 radial cell files per sample. For each representative line, five biological replicates were analyzed.

### Dual-luciferase reporter assay

To assess the promoter activity of *RsOFP2.3*, two promoter fragments (−851 bp and −884 bp relative to the ATG) were amplified from the round-rooted and long-rooted radish genotypes, representing alleles without or with the 312 bp insertion, respectively. These fragments were cloned into the pGreenII0800-LUC vector upstream of the firefly *LUC* reporter gene. The *Renilla luciferase* gene, driven by the CaMV 35S promoter, served as an internal control. The resulting reporter constructs were introduced into *A. thaliana* mesophyll protoplasts *via* polyethylene glycol (PEG)-mediated transfection. LUC activity was measured using the Dual-Luciferase Reporter Assay Kit (Vazyme Biotech, China) in accordance with the manufacturer’s guidelines. For each construct, nine independent biological replicates were analyzed to ensure reproducibility. The primer sequences employed for vector assembly are provided in Table S4.

### Phylogenetic and protein motif analyses of RsTRMs

To identify TRM family members in radish, the amino acid sequences of 34 *A. thaliana* TRM proteins were downloaded from TAIR (https://www.arabidopsis.org/) as previously described [25]. These sequences were used as queries to perform BLASTP searches against the radish genome database (Brassica Database, http://brassicadb.cn) with an *E*-value cutoff of 1e^−10^. After removing redundant sequences, a total of 50 nonredundant RsTRM proteins were obtained. In addition, 11 SlTRM proteins that have been reported to interact with OVATE were retrieved from the public database Sol Genomics Network (SGN, http://solgenomics.net/) [7]. Amino acid sequences of AtTRMs, RsTRMs, and SlTRMs were aligned using ClustalW with default parameters. Phylogenetic trees were inferred in MEGA XI [54] by the neighbor-joining method, and the robustness of each branch was evaluated using 1000 bootstrap replicates.

For conserved motif analysis, the amino acid sequences of 50 RsTRMs and 11 SlTRMs were submitted to MEME [55] to identify conserved motifs. The parameters were set as follows: the number of motifs was limited to 10, motif width ranged from 10 to 100 bp, and site occurrence was restricted between 30 and 120. The relative height of each motif logo was proportional to –log(*P*-value) and was truncated at the threshold corresponding to a *P*-value of 1e^−10^.

### Yeast two-hybrid assay

For Y2H analysis, the coding sequence of *RsOFP2.3* was cloned into the pGBKT7 (BD) vector, while those of *RsTRM4*, *RsTRM12*, *RsTRM15L*, and *RsTRM21* were inserted into the pGADT7 (AD) vector (primer information is listed in Table S4). Co-transformation of bait and prey constructs into the *Saccharomyces cerevisiae* Y2HGold strain (Clontech, USA) was performed as recommended by the manufacturer. Protein interaction was analyzed on synthetic defined (SD) medium deficient in Leu, Trp, His, and Ade.

### Split-luciferase complementation assay

To verify the interaction between RsTRM4 and RsOFP2.3, the coding region of *RsTRM4* without stop codon was fused with the N-terminal fragment of firefly LUC (nLUC) in the pCAMBIA1300 vector, whereas that of RsOFP2.3 was fused with the C-terminal fragment (cLUC) in the same backbone (primer information in Table S4). The recombinant plasmids were introduced into *A. tumefaciens* GV3101, which was subsequently used to infiltrate *N. benthamiana* leaves for transient expression. After an infiltration period of 2–4 days, the leaves were treated with 10 mM D-luciferin (free acid) solution, and bioluminescence was recorded under dark conditions using a low-light, cooled CCD imaging system (NightSHADE LB985, Berthold Technologies) equipped with Indigo software.

### Subcellular localization and bimolecular fluorescence complementation assays

The coding sequences (CDS) of RsOFP2.3 and RsTRM4, excluding their stop codons, were cloned into the pSUPER1300 and pCAMBIA2300 vectors, respectively, to generate the 35S::RsOFP2.3-mCherry and 35S::RsTRM4-GFP constructs. MBD-mCherry as a microtubule marker and CsOVATE-GFP as a cytoplasm marker (a previously reported cytoplasm and nucleus localized protein in cucumber) [30, 56]). The verified constructs were introduced into *A. tumefaciens* strain GV3101 and subsequently infiltrated, either independently or in combination, into leaves of *N. benthamiana*. Infiltrated plants were maintained in the dark for 24 hours and then grown under a 16-hour light/8-hour dark photoperiod for an additional 1–2 days. Visualization of GFP and mCherry fluorescence was performed with a confocal laser scanning microscope (LSM700; Zeiss, Mainz, Germany).

For BiFC analysis, the RsOFP2.3 coding sequence lacking the stop codon was cloned into the pSPYCE(M) vector to generate a fusion with the C-terminal fragment of YFP (residues 156–239). Similarly, the RsTRM4 coding region was inserted into pSPYNE(R) to produce an N-terminal YFP fusion (residues 1–155), following the procedures described by Wu et al. [7] and Waadt et al. [57]. The resulting constructs were introduced into *A. tumefaciens* GV3101 and transiently expressed in *N. benthamiana* leaves. Plants were incubated under the same dark/light regime as above, and YFP signals were subsequently detected using the LSM700 confocal microscope (Zeiss, Mainz, Germany).

## Supplementary Material

Web_Material_uhag127

## Data Availability

The raw sequence data generated in this study have been deposited in the Genome Sequence Archive (GSA: CRA032312) at the National Genomics Data Center, China National Center for Bioinformation/Beijing Institute of Genomics, Chinese Academy of Sciences. The dataset is publicly available at https://ngdc.cncb.ac.cn/gsa.
