# Supplementary material for: A transposable element-derived regulatory variation in RsOFP2.3 underlies morphological diversification of radish taproots through the conserved OVATE family protein-TONNEAU1-recruiting motif module
Source: Hortic Res. 2026 Apr 7;13(8):uhag127. doi: 10.1093/hr/uhag127 (PMC13411279; doi:10.1093/hr/uhag127)
Supplement: Web_Material_uhag127 [file web_material_uhag127.zip › Supplementary Figures M.docx]

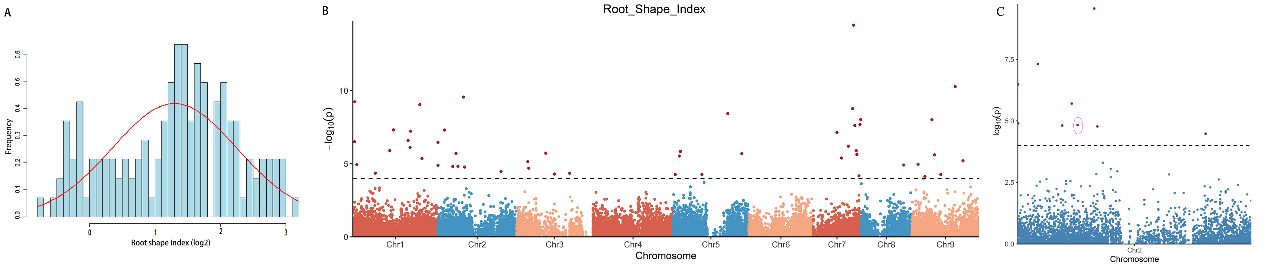


Fig S1. SV-GWAS analysis for taproot shape in radish. (A) Frequency distribution of the taproot shape index in the radish population. Histogram showing the distribution of taproot shape index (log₂-transformed) across the radish accessions. The x-axis represents the log₂ values of taproot shape index, and the y-axis indicates the frequency in the population. The red curve represents the fitted normal distribution. (B) Manhattan plot of SV-GWAS results for taproot shape index. The x-axis represents the physical positions of SVs across the radish genome, and the y-axis shows the –log₁₀(P) values from the association analysis. The horizontal dashed line indicates the genome-wide significance threshold (–log₁₀P = 4.0). (C) Local Manhattan plot of the significant association signal on chromosome 2. The highlighted peak corresponds to a genomic region harboring *RsOFP2.3*, which is significantly associated with taproot shape index.


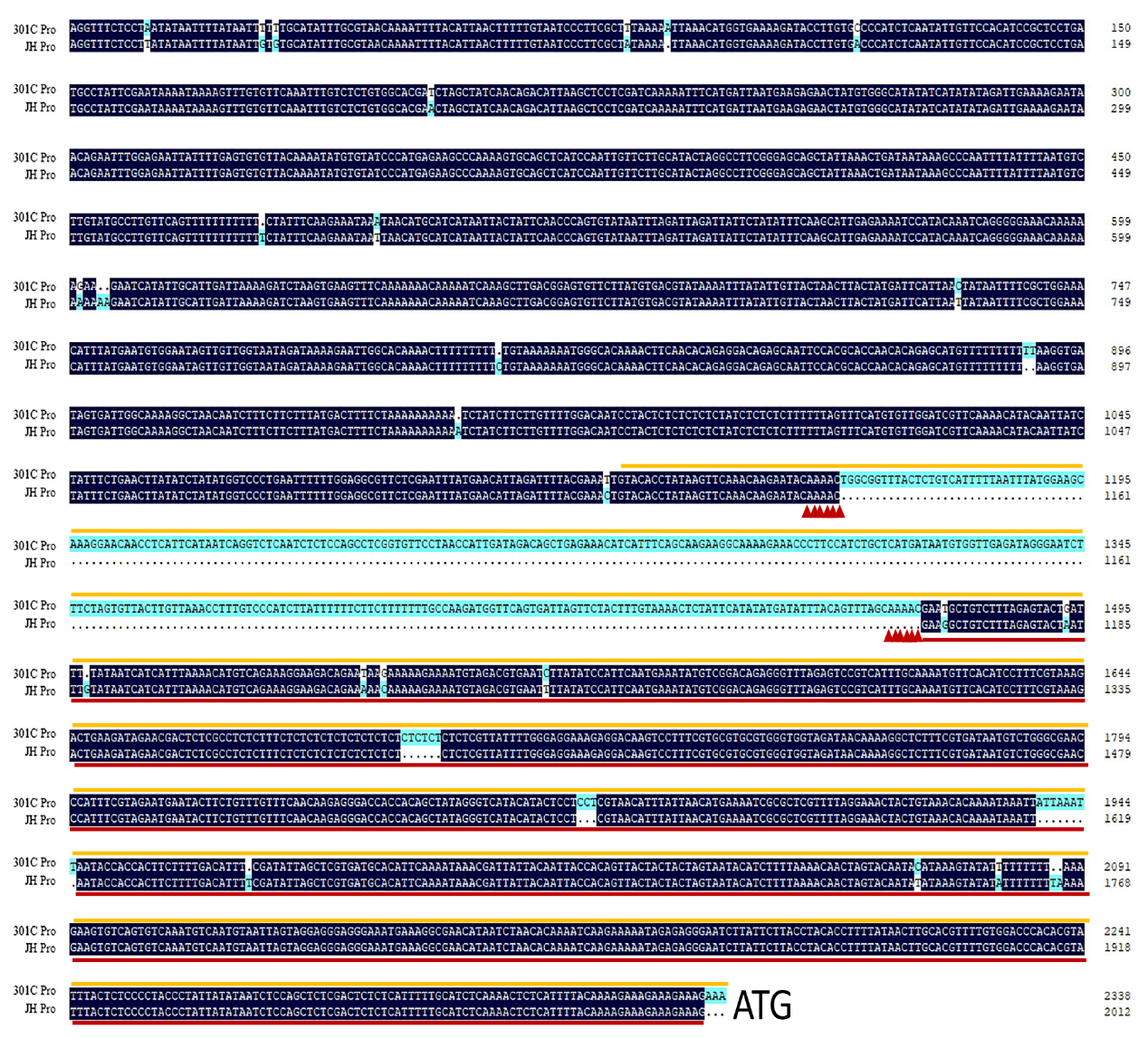


Fig S2. Alignment of *RsOFP2.3* promoter sequences from 301C and JH. The 6-bp direct repeats (CAAAAC) were indicated with red triangle and sequences of P1 and P2 were indicated with orange and red lines, respectively.


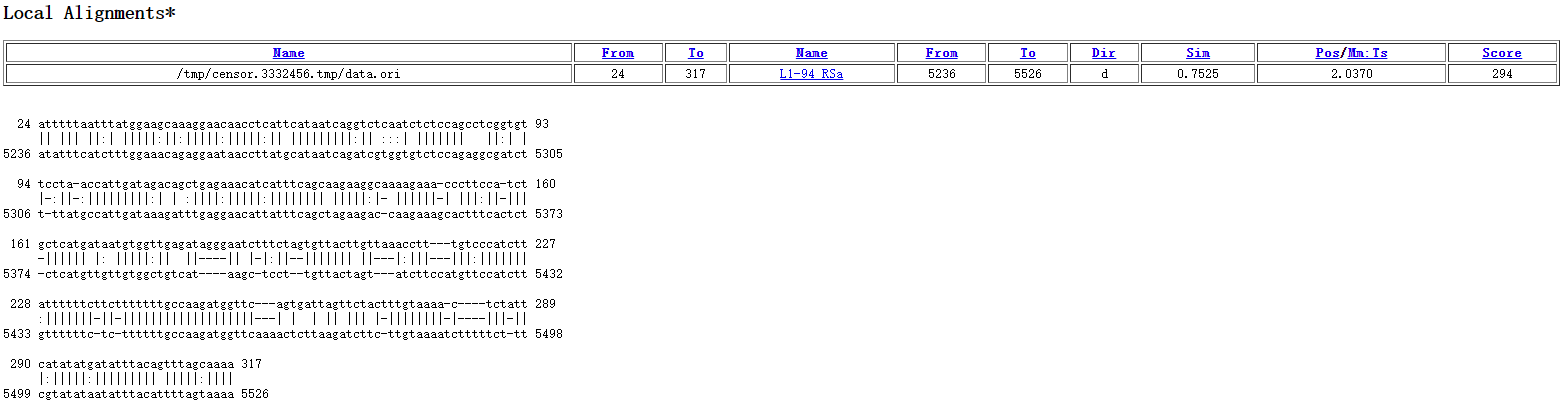


Fig S3. CENSOR alignment of the 306-bp insertion in the *RsOFP2.3* promoter with the retrotransposon L1-94_RSa. The insertion sequence (positions 24–317) was aligned to the *Raphanus sativus* LINE-type non-LTR retrotransposon L1-94_RSa (positions 5236–5526) using CENSOR with the Repbase database. The alignment revealed 75.3% sequence similarity across a 294-bp region (score = 294). Identical nucleotides are marked by vertical bars. This result supports that the insertion represents a truncated fragment of a LINE retrotransposon.


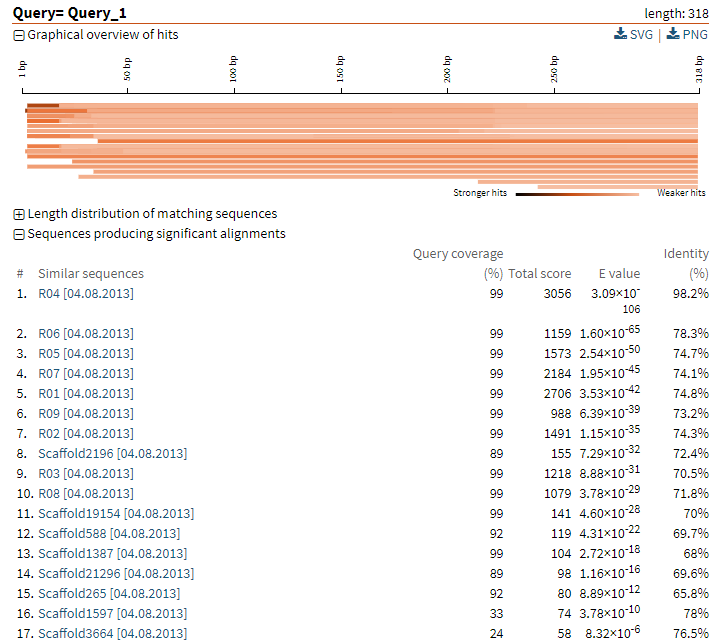


Fig S4. A Blastn search was conducted using the 318 base pairs (comprising a 312 base pair insertion along with one CAAAAC) within the genome database of the Chinese radish cultivar‘Xiangyabai’(http://brassicadb.cn/#/BLAST/). The results showed that multiple copies of the insertion were present in the genome.


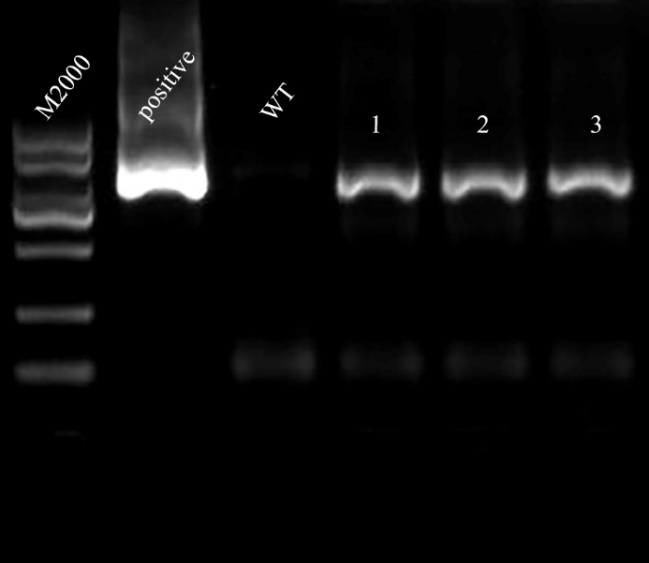


Fig S5. PCR analysis of *RsOFP2.3*-OE transgenic plants with specific primers. Forward sequence in CaMV 35S promoter and reverse sequence in *RsOFP2.3* CDS. The sequence length was 1001 bp.


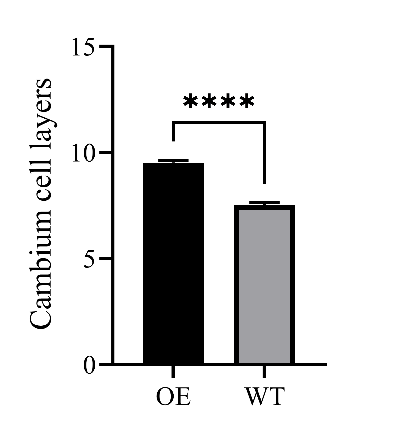


Fig S6. The cell layers of vascular cambium in WT and *RsOFP2.3*-OE taproots. Data are presented as mean±SE. ****P < 0.0001, Student’s t-test.


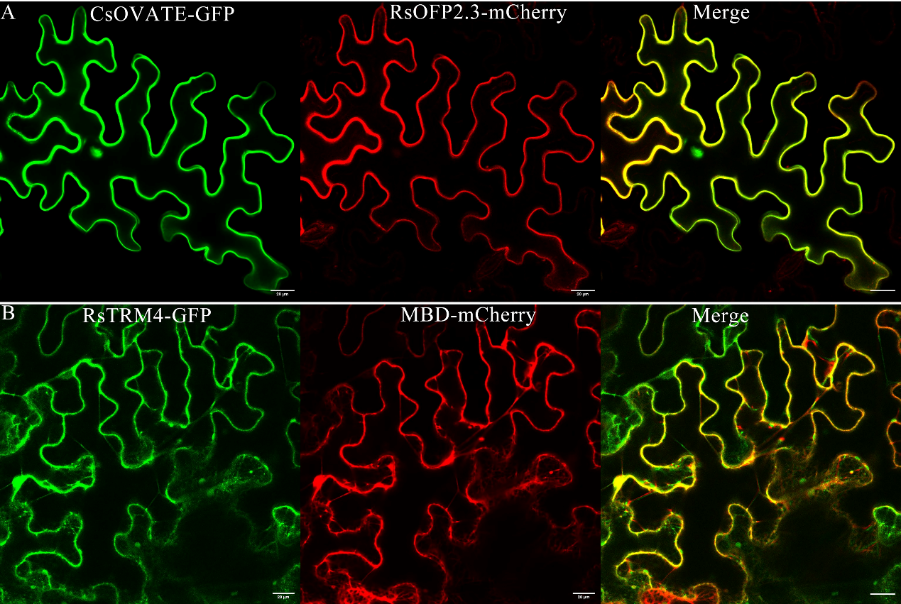


Fig S7. Subcellular localization of RsOFP2.3 and RsTRM4 with marker proteins. (A) Co-expression of RsOFP2.3-mCherry with CsOVATE-GFP, a protein previously reported to localize to both the cytoplasm and nucleus (Wang et al., 2022), in *Nicotiana benthamiana* leaf epidermal cells. (B) Co-expression of RsTRM4-GFP with MBD-mCherry, a microtubule marker, in *N. benthamiana* leaf epidermal cells. Green fluorescence indicates GFP signals, and red fluorescence indicates mCherry signals. Merged images are shown. Scale bars, 20 μm.


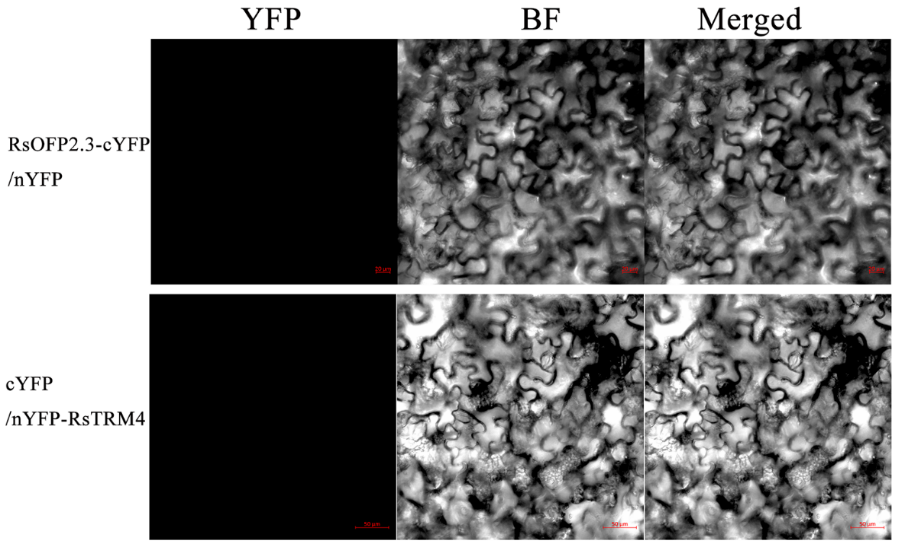


Fig S8. Confirmation of the interaction between RsOFP2.3-cYFP and nYFP-RsTRM4 with empty vectors in *N. benthamiana* leaf epidermal cells using BiFC assay. nYFP and cYFP, N-terminal and C-terminal of YFP, respectively. Scale bar, 20 μm in the upper panel and 50 μm in the lower panel.


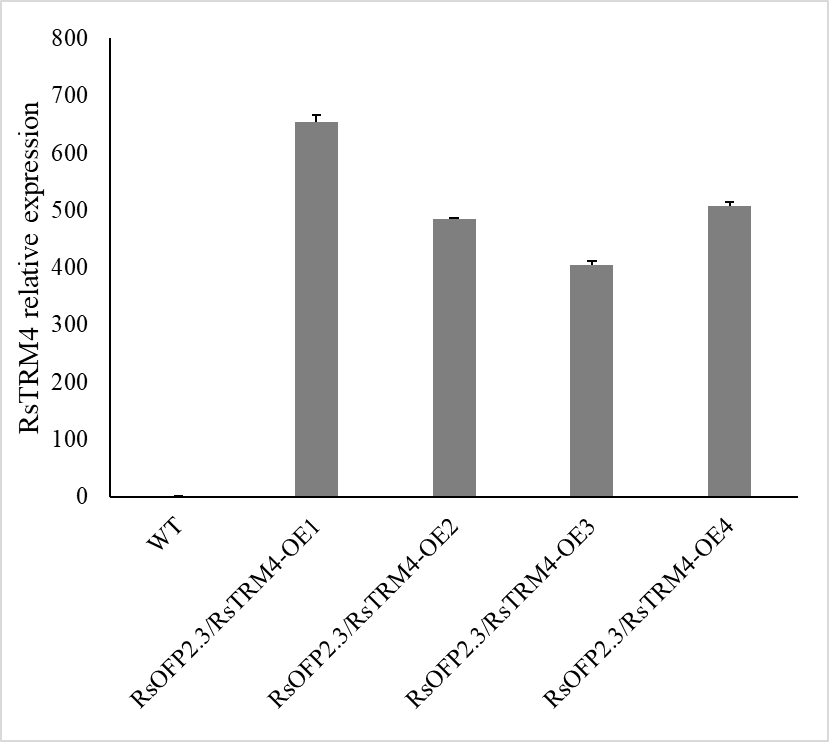


Fig S9. Real-time quantitative RT-PCR analysis showing the overexpression of *RsTRM4* in *RsOFP2.3*-OE representative transgenic lines.


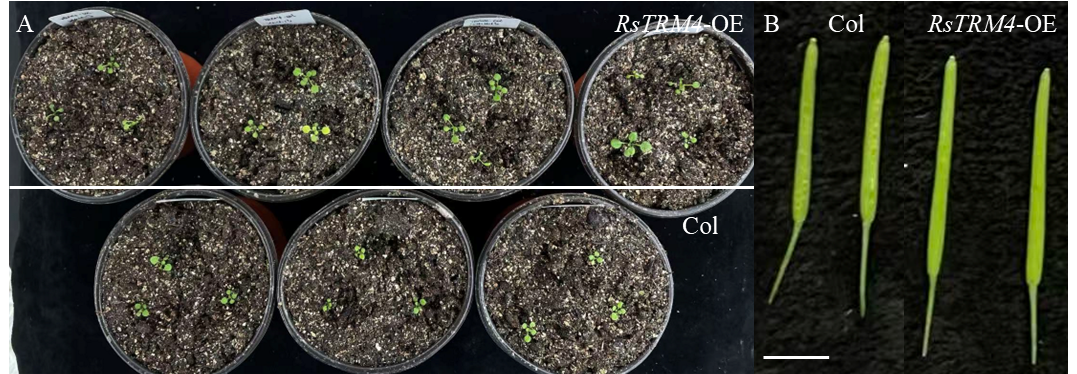


Fig. S10. *RsTRM4* overexpression caused subtle morphological changes compared with wild type. (A) *RsTRM4* overexpression led to petiole elongation at the seedling stage, which disappears with development. (B) *RsTRM4* overexpression led to slight silique elongation. Bar=0.5 cm.
